# Pituitary stalk biopsy – A systematic review of the safety and efficacy of stalk lesion biopsy

**DOI:** 10.1007/s11102-026-01679-5

**Published:** 2026-04-29

**Authors:** Ryan Beerling Dolovac, Ann McCormack, Lachlan R Evans, James A King, Christopher Ovenden, Jeremy Kam, Tony Goldschlager, Mendel Castle-Kirszbaum

**Affiliations:** 1 School of Medicine, University of Western Australia, Perth, Australia; 2 Neuroendocrine Research Group, Applied Medical Research Centre, Department of Endocrinology, St Vincent’s Hospital, Sydney, Australia; 3 St Vincent’s Clinical School, Faculty of Medicine, UNSW, Sydney, Australia; 4 Department of Neurosurgery, The Royal Melbourne Hospital, Parkville, Australia; 5 Department of Surgery, The University of Melbourne, Parkville, Australia; 6 Department of Neurosurgery, Royal Adelaide Hospital, Adelaide, Australia; 7 Faculty of Health and Medical Sciences, Adelaide Medical School, Adelaide, Australia; 8 Department of Neurosurgery, Monash Health, 246 Clayton Road, Clayton VIC 3168 Melbourne, Australia; 9 Department of Surgery, Monash University, Melbourne, Australia

**Keywords:** Pituitary stalk, Infundibulum, Biopsy, Endoscopic, Pituitary, Germ cell tumour, Hypophysitis

## Abstract

**Introduction:**

A myriad of pathological process may involve the pituitary stalk (infundibulum), and despite extensive investigations, biopsy is often required. Due to the rarity of the procedure, the risks and yield of stalk biopsy are not well known, with important implications for clinical decision making.

**Methods:**

A systematic review was performed in accordance with the PRISMA statement. Studies that reported diagnostic yield and complication rates after stalk biopsy were included. Bias was assessed using ROBINS-V2.

**Results:**

A total of 13 studies, including 832 patients and 406 biopsies were included. Mean time from diagnosis to biopsy was 11 months. Preoperative visual symptoms were seen in 29.1%, AVP-deficiency (diabetes insipidus) in 71.0%, and hypopituitarism in 66.3%. Biopsy was predominately performed through the endoscopic transsphenoidal approach (70.9%). Diagnostic yield was 95.8%, and complications included postoperative CSF leak (1.6%), visual worsening (1.6%), new cranial nerve palsy (1.2%), new permanent AVP-deficiency (9.0%), new hypopituitarism (15.7%), and meningitis (2.7%).

**Conclusion:**

Pituitary stalk biopsy is a high yield and safe diagnostic test, with low periprocedural morbidity. The rate of new endocrinopathy compares favourably to the natural history of progressive stalk disease. Stalk biopsy could be considered earlier in the workup of undifferentiated progressive or symptomatic stalk lesions to avoid unnecessary delays in diagnosis and treatment.

**Supplementary Information:**

The online version contains supplementary material available at 10.1007/s11102-026-01679-5.

## Introduction

The pituitary stalk (infundibulum) plays host to a broad spectrum of pathological processes, including neoplastic, inflammatory, and congenital lesions. Stalk lesions often present with endocrinopathy, particularly AVP-deficiency (central diabetes insipidus), but may also cause headache, and visual loss [1]. The breadth of the etiological spectrum necessitates prolonged workup, often across several months. Moreover, significant concerns about the morbidity of stalk biopsy means non-invasive testing is thoroughly exhausted prior to considering surgery. In many cases, stalk biopsy is eventually required to eliminate diagnostic uncertainty and facilitate timely, targeted therapy. In more than half the cases progressing to biopsy, the final histological diagnosis differs from the preoperative differential, highlighting the importance of tissue diagnosis for stalk lesions [2].

Due to the rarity of the procedure, the risks of stalk biopsy have not been well established. It is known that stalk biopsy may be associated with new hypopituitarism and AVP deficiency, as well as the morbidity of the surgical approach, which may either be transsphenoidal or transcranial [2]. However, clear, quantifiable assessment of risks and diagnostic yield is crucial to balanced and informed decision-making when counselling patients with stalk lesions. Here, we aim to systematically analyse the literature on pituitary stalk biopsy to establish evidence-based estimates of risk and diagnostic yield for stalk lesion biopsy.

## Methods

A systematic search of the literature was conducted using the PubMed, Embase, and Scopus databases in accordance with the PRISMA statement. All studies from database inception until November 2025 were queried using the search string: (*pituitary stalk* OR *infundibulum* OR neurohypophys* AND *biopsy*).

Inclusion criteria were: (1) cohort studies or case series; (2) surgeries for biopsy of a stalk lesion; (3) clinical outcomes reported including diagnostic yield and complications. Exclusion criteria included conference abstracts, case reports, studies published in languages other than English, and studies that did not report clinical outcomes or complications. The references of included studies were also individually hand-searched to identify additional eligible studies.

Included studies underwent independent, blinded, data extraction including study year, study size, preoperative hormonal function, surgical technique, diagnostic yield, complication data, and diagnostic pathology. Specific complications extracted where possible included new postoperative AVP-deficiency, postoperative cerebrospinal fluid (CSF) leak, visual worsening, cranial nerve palsy, new hormonal deficiencies, meningitis, mortality, and venous thromboembolic events. Extracted data from studies was tabulated, pooled, and key results summarised in prose.

Risk of bias was assessed using the ROBINS-I-V2 risk of bias assessment scale for non-randomised studies [3].

## Results

Thirteen studies were available for review, comprising 832 patients with disorders of the pituitary stalk [4–16] (Supplementary Fig. 1). All studies were small retrospective cohort studies and case series, and were at serious or critical risk of bias (Supplemental Fig. 2). Studies ranged from 1997 to 2024, with 151.4 years of total study period (Table 1). Of these patients, 406 (48.8%) eventually underwent pituitary biopsy, with a median age ranging from 9 to 37.4, and a slight female predominance (54%). For each centre, the mean biopsy rate was 2.7 per year (range 0.4–15.5 per year).

**Table 1:** Summary of studies of pituitary stalk biopsy

Author	Year	Years of study	Country	*N*	Pituitary Biopsy *N*	Biopsies/Year	Median age	Male : Female
Day et al.	2020	1998–2019 (21 yrs)	USA	9	9	0.43/yr	9.5	6:3
Doknic et al.	2018	2010–2018 (9 yrs)	Serbia	53	8	0.9/yr	NR (32 Mean)	30:23
Imber et al.	2015	1997–2014 (17 yrs)	USA	21	19	1.1/yr	37.4	8:13
Jeon et al.	2022	1994–2018 (25 yrs)	S. Korea	16	16	0.64/yr	9	8:8
Jian et al.	2014	2005–2012 (7 yrs)	China	13	13	1.9/yr	20.0	7:6
Jinguji et al.	2013	2006–2011 (6 yrs)	Japan	11	11	1.8/yr	22	4:7
Kang et al.	2022	2011–2020 (9 yrs)	S. Korea	39	39	4.3/yr	25	15:24
Kinoshita et al.	2017	2002–2015 (13 yrs)	Japan	11	11	0.85/yr	14	7:4
Sawamura et al.	1997	1985–1995 (10 yrs)	Japan / Switzerland	29	16	1.6/yr	18	27:2
Turcu et al.	2013	1987–2006 (20 yrs)	USA	152	37	1.9/yr	NR (44 Mean)	61:91
Wang et al.	2020	2011–2019 (8 yrs)	China	124	124	15.5/yr	18.0	48:76
Zepeda et al.	2024	2020–2022 (2 yrs)	Chile	9	8	4.0/yr	NR (Mean 10.4)	2:7
Zhou et al.	2019	2014–2017 (3.4 yrs)	China	321	95	28.0/yr	NR (31.6 Mean)	143:178
**Total**		**151.4y total**		**832**	**406**	**mean 2.7/yr**		**366:442 (45.3% Male)**

Mean time from diagnosis to biopsy was reported in 3 studies and averaged 11 months (range 3.5–16 months) (Table 2). Preoperative visual loss was seen in 29.1% of patients, while preoperative AVP-deficiency was present in 71.0%. Overall, 66.3% of patients exhibited some form of anterior pituitary hormone dysfunction preoperatively. In the 7 studies analysing each hormonal axis, the gonadotroph (45.2%) and growth hormone (43.3%) axes were deficient more commonly than the thyroid stimulating hormone (31.5%), and adrenocorticotropic hormone (24.0%) axes. Prolactin dysfunction (hyper- or hypo-secretion) was present in 35% of patients.

**Table 2:** Summary of preoperative endocrine function

Author	Mean time diagnosis to biopsy	Preoperative visual loss	Preoperative AVP-Def	Preoperative hypopituitarism	TSH deficiency	ACTH Deficiency	GH deficiency	PRL abnormal	FSH/LH deficiency
Day et al.	NR	0	78% (7)	100% (9)	44% (4)	22% (2)	33% (3)	11% (1)	22% (2)
Doknic et al.	NR	NR	21% (11)	87% (46)	45% (24)	45% (24)	85% (45)	19% (10)	64% (34)
Imber et al.	3.5 months	52% (11)	52% (11)	57% (12)	NR	NR	NR	48% (10)	NR
Jeon et al.	NR	NR	81% (13)	NR	38% (6)	NR	19% (3)	NR	NR
Jian et al.	16 months	7.7% (1)	100% (13)	92% (12)	46% (6)	39% (5)	77% (9)	NR	92% (11)
Jinguji et al.	NR	36% (4)	82% (9)	73% (8)	NR	NR	NR	NR	NR
Kang et al.	NR	36% (14)	44% (17)	74% (29)	NR	NR	NR	NR	NR
Kinoshita et al.	NR	27% (3)	91% (10)	55% (6)	46% (5)	36% (4)	64% (7)	82% (9)	55% (6)
Sawamura et al.	NR	NR	NR	NR	NR	NR	NR	NR	NR
Turcu et al.	NR	11% (16)	28% (43)	32% (49)	21% (32)	15% (23)	18% (28)	19% (29)	29% (44)
Wang et al.	NR	25% (31)	100% (124)	72% (89)	NR	NR	NR	NR	NR
Zepeda et al.	NR	56% (5)	100% (9)	100% (9)	78% (7)	56% (5)	100% (9)	67% (6)	33% (3)
Zhou et al.	12 months	NR	69% (221)	58% (185)	26% (84)	19% (60)	34% (110)	NR	41% (131)
**Total**	**11 months**	**29.11% (69/237)**	**70.97% (445/627)**	**66.28% (405/611)**	**31.48% (136/432)**	**24**,**04% (100/416)**	**43.06% (186/432)**	**34.95% (36/103)**	**44.95% (187/416)**

Biopsies were performed predominantly through an endoscopic transsphenoidal approach (70.9%), with the microscopic transsphenoidal approach (8.9%), open transcranial approach (most commonly a supraorbital craniotomy) (8.5%), neuroendoscopic transventricular approach (8.1%), and stereotactic needle biopsy (3.6%) less commonly implemented (Table 3). Overall biopsy yield was 95.8%. Complications rates after stalk biopsy included postoperative CSF leak (1.6%), visual worsening (1.6%), new cranial nerve palsy (1.2%), new permanent AVP deficiency (9.0%), new hypopituitarism (15.7%), and meningitis (2.7%). No perioperative mortality was described in any study.

**Table 3:** Summary of perioperative outcomes and complications

Author	Biopsy Method	Diagnostic yield	CSF leak	Visual worsening	New CN palsy	New AVP-Def	New hypopituitarism	Meningitis	Mortality
Day et al.	5 ETS, 4 TC	78% (7/9)	0% (0/9)	0% (0/9)	0% (0/9)	11.1% (1/9)	0% (0/9)	0% (0/9)	0% (0/9)
Doknic et al.	NR	100% (8/8)	NR	NR	NR	NR	NR	NR	0% (0/8)
Imber et al.	18 MTS, 1 TC	95% (18/19)	0% (0/19)	0% (0/19)	0% (0/19)	0% (0/19)	19.0% (4/21)	0% (0/19)	0% (0/19)
Jeon et al.	16 NE	94% (15/16)	0% (0/16)	0% (0/16)	0% (0/16)	12.5% (2/16)	18.8% (3/16)	0% (0/16)	0% (0/16)
Jian et al.	12 TC, 1 SNB	100% (13/13)	0% (0/13)	0% (0/13)	0% (0/13)	0% (0/13)	0% (0/13)	0% (0/13)	0% (0/13)
Jinguji et al.	7 ETS, 4 NE	100% (11/11)	9.1% (1/11)	0% (0/11)	0% (0/11)	0% (0/11)	0% (0/11)	0% (0/11)	0% (0/11)
Kang et al.	39 ETS	100% (39/39)	0% (0/39)	0% (0/39)	NR	23.1% (9/39)	33.3% (13/39)	2.6% (1/39)	0% (0/39)
Kinoshita et al.	11 TS	100% (11/11)	0% (0/11)	0% (0/11)	0% (0/11)	0% (0/11)	9.1% (1/11)	0% (0/11)	0% (0/11)
Sawamura et al.	8 SNB, 4 MTS, 4 TC	100% (16/16)	0% (0/16)	0% (0/16)	0% (0/16)	0% (0/16)	0% (0/16)	0% (0/16)	0% (0/16)
Turcu et al.	NR (37 biopsies)	95% (35/37)	NR	NR	2.7% (1/37)	NR	NR	NR	NR
Wang et al.	124 ETS	100% (124/124)	2.4% (3/124)	3.2% (4/124)	1.6% (2/124)	NR	NR	4.8% (6/124)	0% (0/124)
Zepeda et al.	8 TS	100% (8/8)	NR	NR	NR	NR	NR	NR	0% (0/8)
Zhou et al.	NR	88% (84/95)	NR	NR	NR	NR	NR	NR	0% (0/95)
**Total**	**70.9% ETS** **8.9% MTS** **8.5% TC** **8.1% NE** **3.6% SNB**	**95.8% (389/406)**	**1.6% (4/258)**	**1.6% (4/258)**	**1.2% (3/256)**	9.0% (12/134)*	**15.7% (21/134)**	**2.7% (7/258)**	**0.0% (0/369)**

*= 4 additional patients developed transient DI

ETS = Endoscopic transsphenoidal surgery; MTS = microscopic transsphenoidal surgery; TC + transcranial; NE = Neuroendoscopic; SNB = Stereotactic needle biopsy

The most common histopathological diagnosis in patients undergoing stalk biopsy was a germinoma or other germ cell tumour, seen in almost half the patients in the literature (42.3%) (Table 4). Less common diagnoses included hypophysitis (10.7%), Langerhans cell histiocytosis (10.1%), pituitary metastasis (8.2%), Rathke cleft cyst (5.6%), craniopharyngioma (3.9%), glioma (2.1%), and posterior pituitary tumour (e.g. pituicytoma, spindle cell oncocytoma).

**Table 4:** Summary of final histological diagnosis

Pathology	% (*n*)
Germinoma / germ cell tumour	42.3% (217/513)
Langerhans cell histiocytosis	10.1% (52/513)
Hypophysitis	10.7% (55/513)
Craniopharyngioma	3.9% (20/513)
Metastasis	8.2% (42/513)
Glioma	2.1% (11/513)
Posterior Pituitary Tumour	1.4% (7/513)
Rathke’s Cleft cyst	5.6% (29/513)
Other	15.6% (80/513)

Although the included studies focussed on surgical biopsy of stalk lesions, cohorts included lesions managed without biopsy (*n* = 303) [5, 7, 16]. Mean age varied by management strategy and etiology. Stalk biopsy rates were significantly higher in paediatric patients compared to adults (38.5% vs. 26.1%, *p* = 0.028), while patients with congenital lesions (typically managed without biopsy) were significantly younger than those with non-congenital lesions (22.4 vs. 41.5 years, *p* < 0.001) [5, 16]. Endocrine and visual outcomes were not stratified by treatment, precluding comparison.

When stratified by biopsy technique, diagnostic yield remained high across all modalities, while complication profiles differed significantly by approach (Table 5). Transsphenoidal approaches were the most utilised method (*n* = 270) in the literature, with high diagnostic yield (96.3%) balanced against the risks of CSF leak (1.8%) and meningitis (1.1%). Stereotactic needle biopsy showed the highest comparative safety profile, although overall case numbers were more limited (*n* = 45). Transventricular approaches (*n* = 31) carried the highest risk of new permanent AVP-Deficiency (16.1%). Transcranial approaches were rarely utilised (*n* = 14), but were associated with 100% diagnostic yield and minimal morbidity.

**Table 5:** Summary of subgroup outcomes data

Outcome Metric	Transsphenoidal (Endoscopic/Microscopic)	Transventricular (Endoscopic/Open)	Stereotactic Needle Biopsy	Transcranial (Craniotomy)
Total Biopsies (N)	270	31	45	14
Diagnostic Yield	96.3% (260/270)	93.5% (29/31)	97.8% (44/45)	100% (14/14)
CSF Leak	1.8% (5/270)	0.0% (0/31)	0.0% (0/45)	0.0% (0/14)
Meningitis	1.1% (3/270)	0.0% (0/31)	0.0% (0/45)	0.0% (0/14)
New Permanent AVP-Deficiency	4.1% (11/270)	16.1% (5/31)	0.0% (0/45)	0.0% (0/14)
New Anterior Pituitary Deficit	23.7% (64/270)	19.3% (6/31)	0.0% (0/45)	7.1% (1/14)

Stratification by age revealed different pathologies. In the pediatric cohort (*n* = 153), germinomas and germ cell tumors (GCT) were the predominant diagnosis, seen in 68.6% of patients, followed by Langerhans Cell Histiocytosis (LCH) at 19.0%. Notably, no cases of hypophysitis were recorded in children. Conversely, the adult cohort (*n* = 259) was characterized by a higher prevalence of hypophysitis (28.6%), metastatic disease (16.2%), and Rathke’s cleft cysts (10.4%). Preoperative clinical presentation also differed; AVP-deficiency was present in 83.9% of pediatric patients compared to 42.8% of adults. Growth hormone deficiency was similarly more prevalent in the pediatric group (56.8% vs. 23.1% in adults). Despite these differences in pathology, diagnostic yield was nearly identical between children (94.1%) and adults (94.6%).

Diagnosis for non-biopsied groups included congenital anomalies, germinomas, and inflammatory lesions. In 46 cases, the diagnosis was confirmed by either extracranial biopsy (*n* = 45) or positive serum/CSF markers (*n* = 1). In the remaining cases, the diagnosis was presumed based on a benign course or spontaneous remission (*n* = 17), favourable response to radiotherapy (*n* = 9), or typical MRI features (*n* = 23) [5, 15, 16]. Overall, patients managed without biopsy tended to have congenital or self-limiting inflammatory lesions, compared to patients who required biopsy where neoplasms were predominant [7, 14–16].

## Discussion

Pituitary stalk lesions are rare and possess a wide differential diagnosis. In adults, approximately half of lesions are neoplastic, one-third inflammatory, and the remainder congenital. Inflammatory disease is rare in children, and the majority of lesions are neoplastic (70%) or congenital (20%) [17]. Workup is typically prolonged, with an 11-month average delay from diagnosis to biopsy in our study. Serum testing for inflammatory disorders and tumour markers, cerebrospinal fluid analysis, and whole body screening imaging are considered first line investigations in the setting of an isolated stalk lesion [18]. However, a significant proportion of cases eventually require tissue diagnosis due to lesion growth, diagnostic uncertainty, or progressive hormonal and visual failure [19].

Stalk biopsy is the gold standard diagnostic test, but indications are controversial [18]. Stalk thickness is often used as an indication for biopsy, but cutoffs are varied. The normal stalk measures approximately 2 mm at its insertion into the pituitary gland and 3 mm at the level of the optic chiasm [20]. Consensus guidelines thus define a thickened stalk as ≥3 mm at the pituitary insertion and ≥4 mm at the base of the infundibular recess [21, 22]. As stalk thickness increases, so does the likelihood of a neoplastic process [19, 23, 24], with a cutoff of > 5.3 mm highly predictive of tumour infiltration [23]. The morphology of the stalk thickening may also be useful in the differential diagnosis. Neoplasms tend to have extrasellar involvement, and asymmetric, nodular stalk thickening, while inflammatory processes involve the entire stalk diffusely [23]. Up to 20% of initially indeterminate cases demonstrate progression on serial imaging [19], hence biopsy is often required after a period of observation. Progression of stalk thickening over time is associated with neoplastic processes, rather than hypophysitis [24], although radiologically perceptible progression may take many years [25–27].

Stalk biopsy is often delayed due to concerns of surgical morbidity, particularly worsening endocrinopathy and visual loss. Our study has shown that when performed in experienced centres, predominantly through an endoscopic transsphenoidal approach, surgical morbidity is low. AVP-deficiency is seen in two-thirds of patients preoperatively, and new permanent AVP-deficiency occurs in 9% of patients after surgery. Hypopituitarism is seen in nearly half of patients preoperatively, and at least one axis demonstrates a new deficiency in 16% of patients after surgery. However, the natural history of stalk neoplasms is that of progressive endocrinopathy, with 57% developing a new anterior pituitary deficiency during follow up [19]. Upfront endocrine risk with biopsy is thus low, particularly when considering the natural history of progressive endocrine failure. Moreover, earlier initiation of targeted therapy facilitated by biopsy may reverse endocrinopathy [28]. Overall procedural risks were low, with the rate of visual deterioration and cranial neuropathy both in the order of 1–2%. No perioperative deaths were reported in over 400 cases. All surgical techniques provided high (> 93%) diagnostic yield. The side effect profile of each technique was characteristic of the approach, with higher rates of CSF leak and meningitis with transsphenoidal approaches. Endocrine outcomes were broadly similar across techniques, with a trend for higher rates of new anterior pituitary hormone deficiency with endonasal biopsy. These differences may also reflect variation in lesion anatomy (infundibulo-sellar vs. pure infundibular vs. infundibulo-hypothalamic) and lesion type (exophytic vs. homogenously enlarged) that is incorporated into surgical decision making.

The endoscopic endonasal approach (EEA) has been adopted as the preferred route to the central skull base, and is the most common technique for stalk biopsy in modern series. Benefits of the EEA are a direct, subchiasmatic view of the of stalk, avoiding the need for optic apparatus or large vessel manipulation, and high magnification and illumination facilitating targeted biopsy of pathological tissue while avoiding uninvolved stalk and hypothalamus. In select cases with intrasellar extension, the procedure is entirely extra-arachnoid, but in the majority of cases, suprasellar arachnoid is breached and watertight skull base reconstruction is required. Postoperative CSF leak was uncommon, seen in only 1.6% of cases. For lesions not suitable for the EEA, such as in the setting of poorly pneumatised sinuses or isolated lesions of the 3rd ventricular floor, neuroendoscopic transventricular approaches [7], subfrontal craniotomy [8], and stereotactic needle biopsy [12] have all been used successfully. Considerations to improve diagnostic yield include the use of frameless stereotaxy with up-to-date imaging to corroborate intraoperative visualisation of lesional anatomy and biopsy location, an appropriate duration preoperatively without high dose exogenous steroid or other immunomodulators when sampling inflammatory lesions, and accounting for sampling error when biopsying neoplastic lesions (particularly GCT) that may have mixed histology.

Overall diagnostic yield across all techniques was 95.8%, and germ cell tumours and inflammatory disorders formed the bulk of the pathological diagnoses. Correct tissue diagnosis is vital to guide therapy and reduce the risk of iatrogenic complications from erroneous empiric medical and radiation therapy. More than half of stalk biopsies lead to a change in the presumptive preoperative diagnosis [10], emphasising the importance of tissue diagnosis in progressive cases with any diagnostic uncertainty. Similarly, in this era of targeted therapy, molecular analysis of stalk lesions afforded by biopsy may determine eligibility for targeted therapies and clinical trials. Examples include B-cell targeted immunomodulators for IgG4 related disease [29], BRAF-MEK inhibition for papillary craniopharyngioma [30] and Langerhans cell histiocytosis [31], and MEK and VEGF inhibitors for suprasellar glioma [32]. Given the low periprocedural morbidity and acceptable rate of new endocrinopathy with stalk biopsy, earlier referral may be useful to avoid unnecessary diagnostic delays, provide definitive diagnostic answers, and facilitate institution of targeted therapies where appropriate.

Our study has several limitations. Included studies were retrospective with small sample sizes and at high risk of bias, reflecting the rarity of these lesions. Not all studies reported hormonal outcomes or surgical morbidity, and results were not stratified by age, surgical technique, stalk morphology, or lesion type. Indications for biopsy also differed between centres, introducing further heterogeneity and limiting generalisability. Rates of new AVP-deficiency and anterior pituitary dysfunction were incompletely reported, including the specific hormonal axes affected. Comparisons of endocrine outcomes between surgical approaches could not be controlled for differences in patient age, lesion anatomy, surgeon experience, extent of resection, and underlying pathology, limiting their utility. Finally, this study focused on surgical series, which inherently reflect a selection bias toward symptomatic or growing stalk lesions. Analysis of patients managed without biopsy demonstrated this group were generally younger, had lesions that were predominantly congenital, stable across serial imaging, or exhibited spontaneous remission [16]. Thus, although our data suggest stalk biopsy is safe and high yield, it should still be reserved for growing, symptomatic lesions with diagnostic uncertainty despite thorough workup.

## Conclusions

Pituitary stalk biopsy is a high yield and safe diagnostic test for lesions of the infundibulum. Periprocedural morbidity is low (< 2%) and the rate of new endocrinopathy compares favourably to the natural history of progressive stalk disease. Given its safety and efficacy, endoscopic endonasal stalk biopsy could be considered earlier in the workup of undifferentiated growing, symptomatic stalk lesions that have exhausted non-invasive testing to avoid unnecessary delays in diagnosis and treatment.

## Electronic Supplementary Material

Below is the link to the electronic supplementary material.

Supplementary Material 1

## Data Availability

All data analysed and reported in this paper comes directly from the cited articles, with all information being publicly available.

## Code availability

N/A.
