# Supplementary material for: Pituitary stalk biopsy – A systematic review of the safety and efficacy of stalk lesion biopsy
Source: Pituitary. 2026 Apr 29;29(3):77. doi: 10.1007/s11102-026-01679-5 (PMC13124891; doi:10.1007/s11102-026-01679-5)

**Supplementary Figures**

Supplementary Figure 1 – PRISMA flow chart of the systematic review process


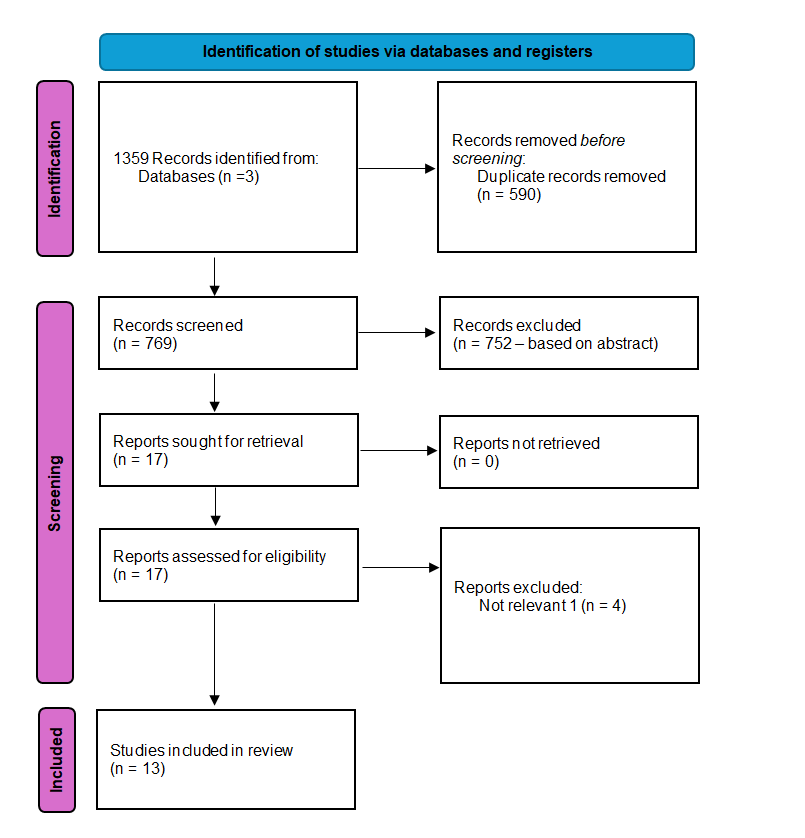


Supplementary Figure 2 – Risk of bias assessment of included studies using the ROBINS-V2 tool


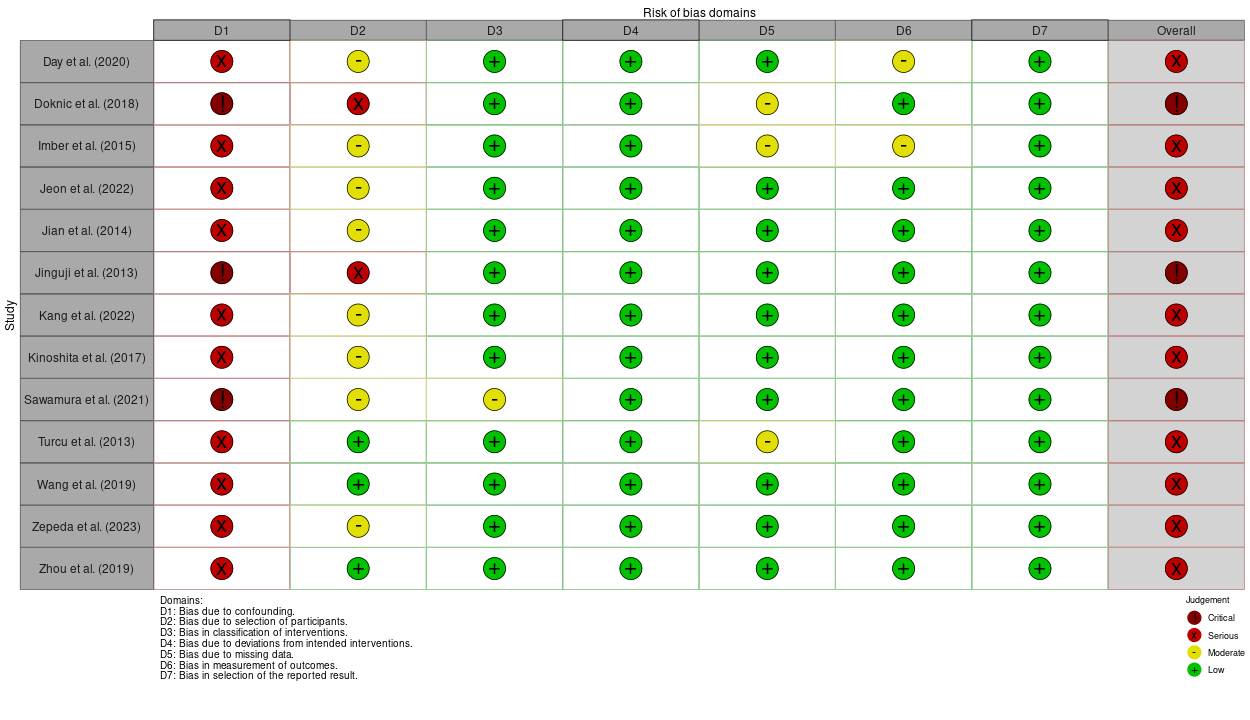

Supplement: Supplementary file 1 — Supplementary Material 1 [file 11102_2026_1679_MOESM1_ESM.docx]
